# The pemetrexed-containing treatments in the non-small cell lung cancer, is -/low thymidylate synthase expression better than +/high thymidylate synthase expression: a meta-analysis

**DOI:** 10.1186/1471-2407-14-205

**Published:** 2014-03-19

**Authors:** Lei Wang, Rui Wang, Yunjian Pan, Yihua Sun, Jie Zhang, Haiquan Chen

**Affiliations:** 1 Department of Thoracic Surgery, Shanghai Medical College, Fudan University Shanghai Cancer Center, 270 Dong-An Road, Shanghai 200032, China; 2 Department of Oncology, Shanghai Medical College, Fudan University Shanghai Cancer Center, Shanghai, China

**Keywords:** Thymidylate synthase, Pemetrexed, Lung cancer, Meta-analysis

## Abstract

**Background:**

The predictive value of thymidylate synthase (TS) for clinical sensitivity to pemetrexed-containing chemotherapy in patients with non-small cell lung cancer (NSCLC) remains controversial. This meta-analysis is performed to provide an assessment of whether expression variations of TS are associated with objective response in patients with NSCLC treated with pemetrexed-containing chemotherapy.

**Methods:**

An electronic search was conducted using the databases MEDLINE, EMBASE and CNKI, from inception to June 10^th^, 2013. A systemic review of the studies on the association between TS expression in NSCLC and objective response of pemetrexed-containing regimen was performed. Pooled odds ratios (OR) for the response rate were calculated using the software Revman 5.0.

**Results:**

There were a total of 526 patients in the eight studies that met our criteria for evaluation. +/high expression of TS was found in 269 patients (51.1%), and -/low expression for this gene was found in 257 (48.9%) patients. The objective response rate for pemetrexed-containing chemotherapy was significantly higher in patients with -/low expression TS expression (OR = 0.45; 95% CI, 0.29–0.70; *p* = 0.0004). Although patients with -/low expression of TS have a longer median overall survival time and progression free survival time than those with +/high expression of TS, the difference was not statistically significant.

**Conclusions:**

*−*/low expression of TS was associated with higher objective response in NSCLC patients treated with pemetrexed-containing chemotherapy. TS may be a suitable marker of sensitivity to pemetrexed-based chemotherapy in patients with NSCLC.

## Background

Lung cancer is the most-common cause of cancer-related mortality worldwide and non-small cell Lung cancer (NSCLC) accounts for more than 85% of primary lung cancers and approximately two-thirds of NSCLC patients are diagnosed at an advanced stage [1-3]. Platinum-based chemotherapy is appropriate for selected patients who have a good performance status [4,5]. But the approach of treating patients with a platinum-containing regimen may have reached a plateau in terms of efficacy [6]. Most patients receiving front-line chemotherapy may experience disease progression and need second-line therapy [7]. One of several treatments for NSCLC as the second line therapy is pemetrexed, which is increasing its therapeutic scope from second-line therapy to first-line and maintenance therapy [8-10].

Pemetrexed is a multitargeted antifolate agent, inhibiting at least three of the enzymes involved in DNA synthesis and folate metabolism: thymidylate synthase (TS), dihydrofolate reductase (DHFR) and glycinamide ribonucleotide formyl transferase (GARFT) [11,12]. Among them, TS is a key enzyme that catalyzes the methylation of fluorod UMP, the precursor of DNA synthesis, into dTMP [13]. Recent in vitro studies demonstrated that lung cancer cell lines with low thymidylate synthase expression were highly sensitive to pemetrexed [14-16]. Thymidylate synthase expression in NSCLC has attracted a considerable attention because of its potential role as a promising predictor for response to pemetrexed-based chemotherapy.

A number of studies have explored the relationship between thymidylate synthase expression and overall response rate in NSCLC patients, but clinical data about TS expression and its predictive value in NSCLC patients receiving pemetrexed -containing chemotherapy are still inconclusive. There are published reports supporting that significantly higher response rates were associated with TS-negativity compared with TS-positivity in patients with NSCLC especially in those with nonsquamous NSCLC treated with pemetrexed-based Chemotherapy [3,17,18]. However, there are also reports of studies failed to find such an association [6,19-22]. Recently, several studies have demonstrated that high level of TS expression was associated with poor prognosis, suggesting that TS expression may be useful to predict survival after complete resection in p-stage I adenocarcinoma of the lung [23-25]. Whereas Zheng and colleagues found that patients with high TS expression actually had significantly increased overall survival (OS) when compared to patients with low expression [26]. To determine whether TS expression is associated with objective response in NSCLC patients treated with pemetrexed-containing therapy, we reviewed published studies and carried out a meta-analysis.

## Methods

### Search strategy

The search was performed by consulting the electronic database MEDLINE, EMBASE and CNKI f or all relevant papers published from the earliest publication date included in the database onward to June 10^th^ 2013. Searches included the terms TS OR thymidylate synthase and lung cancer. The results were then hand searched for eligible studies. No language restrictions were imposed. The references of retrieved articles were also screened for relevant articles and two authors (L Wang and R Wang) conducted all searches independently.

### Eligibility criteria

The following criteria for eligibility among studies were set before collecting articles: (1) utilized pemetrexed –containing chemotherapy for patients with pathologically proven NSCLC. (2) measured TS with immunohistochemistry (IHC) or real-time reverse transcriptase PCR (RT-PCR); (3) presented the data of objective response according to TS status.

### Quality assessment

Two investigators (L Wang and J Zhang) independently assessed the quality of each study using the Newcastle–Ottawa Quality Assessment Scale. Discrepancies were resolved by consensus. The Newcastle–Ottawa Quality Assessment Scale involves assessing three categories – patient selection, study comparability and outcome–based on eight items. Stars awarded to high-quality elements are used to compare study quality in a qualitative manner. Four items in the selection category, two items in the comparability and three items in the outcome category; a maximum of two stars can be given for comparability; a study can be awarded one star for each item in these categories. The scoring system was recommended by the Cochrane Non-randomized Studies Methods Working Group [27,28].

### Statistical analysis

Two independent reviewers extracted the required information using pre-determined forms. Data on objective response rate were analyzed. The data were entered into the Cochrane Collaboration software (RevMan Version 5.0 for Windows; the Cochrane Collaboration, Oxford, UK) and the Cochran’s test was used to assess the heterogeneity of included studies. For the heterogeneity tests, a *P*-value below 0.05 was considered to indicate significance. If the test of heterogeneity was significant (*p* < 0.05, *I*^2^ > 50%) the random-effect model would be used, otherwise the fixed model would be used. Publication bias was estimated by examining the relationship between the treatment effects and the standard error of the estimate (S.E log OR) using a funnel plot. Several additional sensitivity analyses (chemotherapy regimens and TS measurement methods) were also performed to further detect and evaluate clinical heterogeneity.

## Results

### Selection of studies

**Figure 1:**
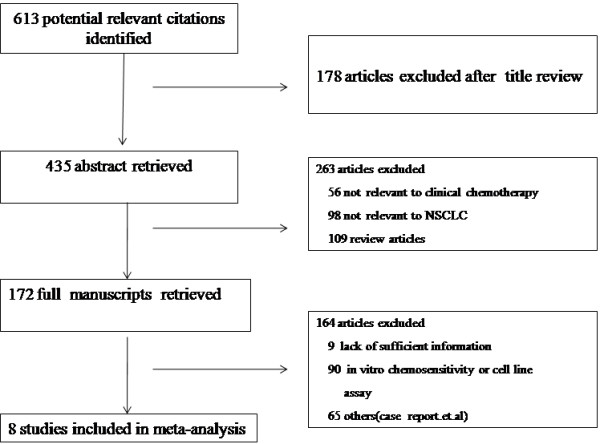
Flow chart of article selection in this meta-analysis: eight studies involving 526 patients were analyzed.

The search strategy identified 613 potentially relevant articles, 178 of which were excluded after the titles were reviewed. A total of 435 studies were included for abstract review after the first exclusion. Among the 435 studies, 56 of them were not relevant to clinical chemotherapy (only illustrating the TS expression and its clinicopathological correlation to NSCLC) and 98 of them were not relevant to NSCLCS (studying TS in small cell lung cancer, neuroendocrine, malignant pleural mesothelioma, thymic tumors, primary colorectal cancer, and so on). There were also 109 review articles in the 435 studies above. So 263 articles were excluded and 172 studies were extracted for full text review after careful abstract review. We excluded 164 studies due to lack of sufficient information or methods discrepancies. After completing the selection process, data from a total of eight studies involving 526 patients (Figure 1) systematically was analyzed. All of them studied the association between TS expression and response to pemetrexed-containing chemotherapy [6,22-31].

**Figure 2:**
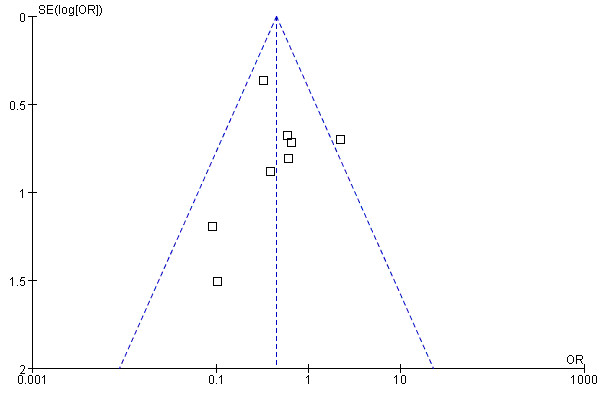
Funnel plot for the detection of publication bias.

The Newcastle–Ottawa Scale, composing of eight items that assess patient selection, study, comparability and outcome, was used to conduct the quality assessments for the eight studies. This scale has been adopted in other non-randomized studies [28,32,33]. Studies which met five or more of the eight criteria were given higher quality scores. A summary of the studies which scored highly is shown in Table 1. Characteristics of the eligible studies are presented in Table 2. Bias assessment was evaluated by funnel plot analysis shown in Figure 2 and the heterogeneity in the 8 studies was not significant statistically (*p* = 0.23).

**Table 1:** Quality of the studies used in the meta-analysis

**Studies**	**Selection(stars)**	**Comparability(stars)**	**Outcome(stars)**
Bepler et al. [6]	4	2	3
Chang et al. [30]	3	2	3
Chen et al. [21]	4	2	3
Igawa et al. [31]	3	2	3
Park et al. [22]	3	2	3
Sun et al. [17]	4	2	3
Takezawa et al. [18]	3	2	3
Wang et al. [19]	3	2	3

The Newcastle–Ottawa Quality Assessment Scale is composed of three categories, selection, comparability and outcome. Stars are awarded to high-quality elements and are used to compare study quality in a quantitative manner. There are four items in the Selection category and three items in the Outcome category; a study can be awarded one star for each item in these categories. A maximum of two stars can be given for comparability.

**Table 2:** Characteristics of studies included in the meta-analysis

**Study**	**All pts**	**TS detection method**	**Chemotherapy regimen**	**Ethnicity**	**TS expression**	**Evaluable for response**	**Disease stage**	**ECOGPS**	**TS high/+**		**TS low/-**	
									**OR (pts)**	**Total pts**	**OR (pts)**	**Total pts**
Bepler et al. [6]	52	RT-PCR	pemetrexed + gemcitabine	Caucasian	35	35	I-III	NR	5	17	7	18
Chang et al. [30]	110	IHC	pemetrexed	Asian	55	52	advanced or reoccurent	0-4	23	41	4	11
Chen et al. [21]	268	IHC	pemetrexed	Asian	49	42	IIIB-IV	NR	3	20	5	22
Park et.al [22]	98	IHC	pemetrexed	Asian	98	88	IB,IIIA-IV	NR	5	54	5	34
Takezawa et al. [18]	24	IHC	pemetrexed + platinum	Asian	24	24	IIIB-IV	0-1	1	12	6	12
Wang et al. [19]	38	RT-PCR	pemetrexed + platinum	Asian	38	38	IIIB-IV	0-1	2	10	11	28
Igawa et al. [31]	104	IHC	pemetrexed	Asian	54	54	IIIB-IV or reoccurent	0-3	0	23	5	31
Sun et al. [17]	285	IHC	pemetrexed	Asian	149	149	IIIB-IV	0-1	9	75	21	74
			pemetrexed + platinum	Asian	44	44	IIIB-IV	0-1	4	17	13	27

*Abbreviations*: *TS* thymidylate synthase, *NR* no report, *IHC* immunohistochemistry, *RT-PCR* real-time reverse transcriptase PCR, *NSCLC* non-small cell lung cancer, *PTS* patients.

### Objective response

**Figure 3:**
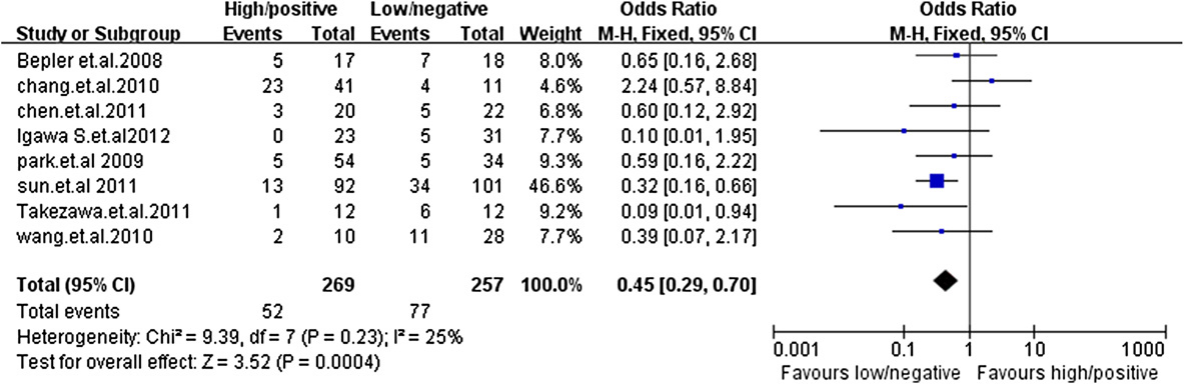
**Comparison of objective response rate between the TS -/low arm and TS +/high arm of NSCLC patients receiving pemetrexed-containing chemotherapy (n = 526).** Pooled data from these 8 studies showed an overall objective response rate of 19.3% and 30.0% for TS +/high expression (n = 269) and TS -/low expression (n = 257), respectively, which was significantly in favor of TS -/low expression (OR = 0.45; 95% CI, 0.29–0.70; *p* = 0.0004).

All studies reported data on tumor objective response (Table 2), which included complete and partial tumor responses, stable disease and progression disease. Because no heterogeneity was found across studies (Chi^2^ = 9.39, *p* = 0.23; *I*^2^ = 25%), the fixed-effects model was used. Pooled data from these eight studies showed an overall objective response rate of 19.3% for TS +/high expression (n = 269) and 30.0% for TS -/low expression (n = 257). These results indicate a statistically significant favorable clinical outcome for patients with -/low expression TS. The pooled odds ratio from the eight studies was 0.45 (OR = 0.45; 95% CI, 0.29–0.70; *p* =0.0004; Figure 3).

**Figure 4:**
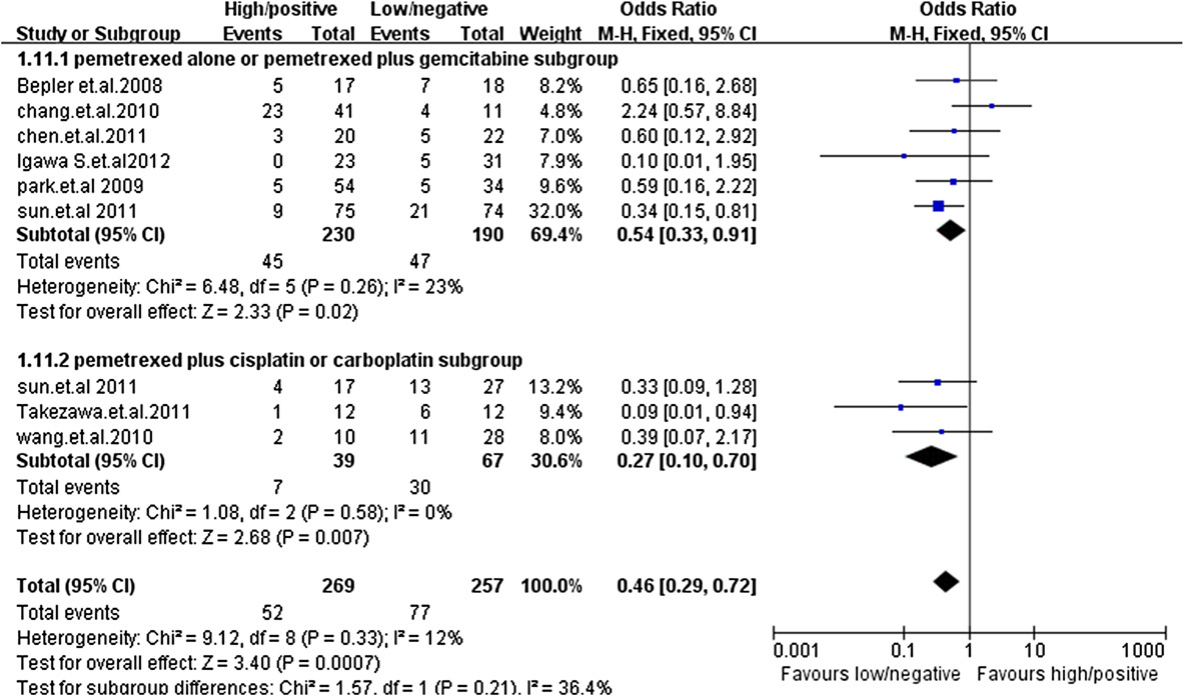
**Subgroup analysis by chemotherapy regimen.** In the subgroup of the patients treated with pemetrexed plus cisplatin or carboplatin, overall objective response rate in TS -/low patients was significantly higher than that in TS +/high patients (OR = 0.27, 95% CI 0.10–0.70, *p* = 0.007). For the patients treated with pemetrexed monotherapy or pemetrexed plus gemcitabine, the overall objective response rate for patients with -/low TS expression was significantly higher than that for patients with +/high TS expression(OR = 0.54, 95% CI 0.33–0.91, *p* = 0.02).

Subgroup analysis was conducted based on chemotherapy regimen. Six studies described clinical results with patients treated with pemetrexed monotherapy or pemetrexed plus gemcitabine and three clinical studies described results of patients treated with pemetrexed plus cisplatin or carboplatin. For the regimen of pemetrexed monotherapy or pemetrexed plus gemcitabine, the overall objective response rate for patients with -/low TS expression was higher than that for patients with +/high TS expression(OR = 0.54, 95% CI 0.33–0.91, *p* = 0.02). For patients treated with pemetrexed plus cisplatin or carboplatin, a significantly better objective response rate was also observed in patients with -/low TS expression (OR = 0.27, 95% CI 0.10–0.70, *p* = 0.007). There existed no heterogeneity between two treatment subgroups (*I*^2^ = 12%) (Figure 4). Although the objective response rate difference did not reach statistical significance between patients treated with platinum-free and platinum containing subgroup, −/low TS expression patients treated with pemetrexed plus cisplatin or carboplatin might be more likely to achieve complete or partial response. (Figure 4).

**Figure 5:**
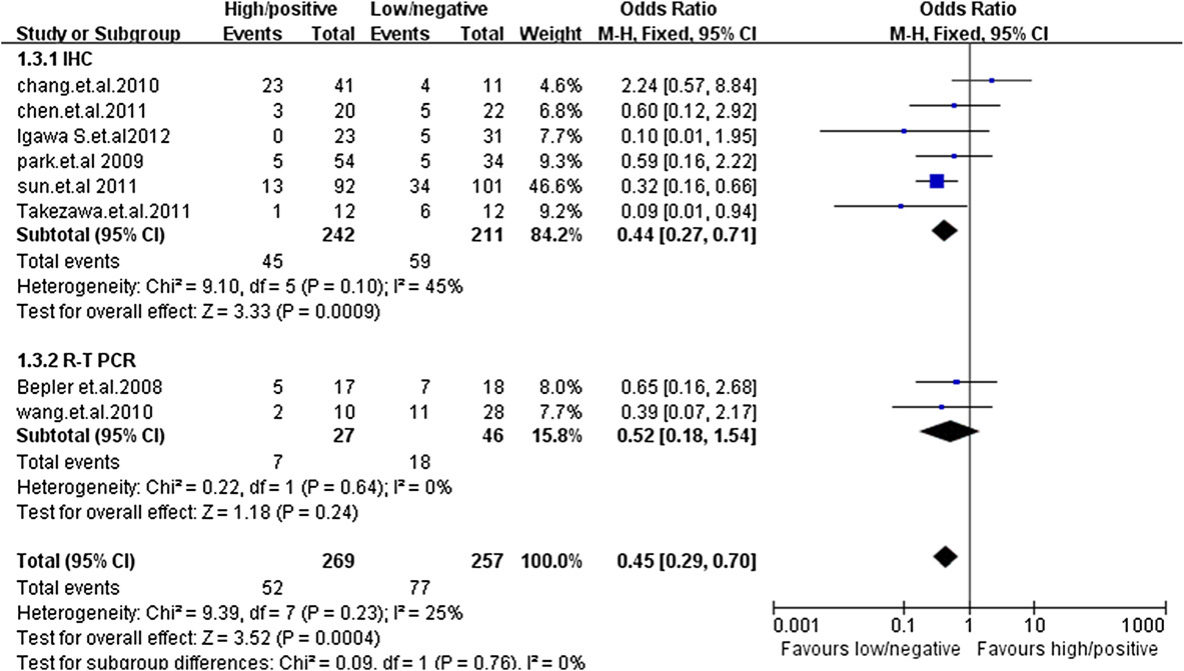
**Subgroup analysis by TS detection method.** In IHC subgroup, objective response rate in TS -/low expression patients was significantly higher than that in TS +/high expression patients (OR = 0.44; 95% CI, 0.27–0.71; *p* = 0.0009). And in Real-time reverse transcriptase PCR subgroup, there was a trend that TS -/low predicted better objective response rate, but the difference did not reached statistical significance (OR = 0.52; 95% CI,0.18-1.54 *p* = 0.24).

Immunohistochemistry (IHC) was used in 6 studies and real-time reverse transcriptase PCR(RT-PCR) was used to detect TS in 2 studies and. In IHC subgroup, objective response rate in TS -/low expression patients was significantly higher than that in TS +/high expression patients (OR = 0.44; 95% CI, 0.27–0.71; *p* = 0.0009). In Real-time reverse transcriptase PCR subgroup, there was a trend that TS -/low predicted better objective response rate, but the difference did not reached statistical significance (OR = 0.52; 95% CI,0.18-1.54 *p* = 0.24). We noted no evidence of heterogeneity between TS detection method subgroups in this meta-analysis (Chi^2^ = 8.33, *p* = 0.22; *I*^2^ = 28%) (Figure 5).

### Median survival time and time to progression

Median survival data were available in five of 8 studies on the association between TS expression and response to pemetrexed-containing chemotherapy [6,17,21,30,31]. Patients with +/high expression of TS had a median overall survival(OS) time of 14.4 months; patients with -/low expression of TS had a median overall survival time of 19.0 months. Although those with -/low expression of TS have a longer median overall survival time than those with +/high expression of TS, the difference was not statistically significant (*p* = 0.239) (Table 3). Similar association was also found in five studies that provide sufficient data for progression free survival time(PFS) (7.6 months in patients with -/low expression of TS Vs 5.8 months in patients with +/high expression of TS, *p* = 0.13) [6,17,21,30,31] (Table 4).

**Table 3:** Median overall survival time in the studies

**Study**	**Median survival time(months)**		**Median ratio**	***p *** **value**
	**TS +/high**	**TS -/low**		
Bepler et al. [6]	27.8	27	0.97	0.272
Chang et al. [30]	6.7	9.5	1.42	0.688
Chen et al. [21]	10	21.4	2.14	0.09
Sun et al. [17]	18.9	22.3	1.18	0.106
Igawa et al. [31]	8.6	14.7	1.71	0.04
Pool	14.4	19.0	1.32(95% CI 0.92-2.72)	0.24

Patients with +/high expression of TS who received pemetrexed-based chemotherapy had a median overall survival time of 14.4 months; patients with -/low expressionof TS had a median overall survival of 19.0 weeks. Although patients with -/low expression of TS perform a longer median overall survival time than those with +/high expression of TS, the difference was not statistically significant (*p* = 0.24).

**Table 4:** Median progression free survival time in the studies

**Study**	**Median progression free survival time(months)**		**Median ratio**	***p *** **value**
	**TS +/high**	**TS -/low**		
Bepler et al. [6]	20.7	21	1.01	0.187
Chang et al. [29]	1.3	2.4	1.85	0.407
Chen et al. [21]	3.4	4.8	1.41	0.01
Sun et al. [17]	2	4.1	2.05	0.001
Igawa et al. [30]	1.6	5.8	3.63	0.03
Pool	5.8	7.6	1.31 (95% CI 0.43-2.19)	0.13

Patients with +/high expression of TS who received pemetrexed-based chemotherapy had a median progression free survival time of 5.8 months; patients with -/low expressionof TS had a median progression free survival time of 7.6 months. Although there was a trend that TS -/low predicted better progression free survival time, the difference did not reached statistical significance (*p* = 0.13).

## Discussion

In this meta-analysis, we evaluated the effects of expression variations in TS on the objective response rate to pemetrexed-containing chemotherapy for NSCLC. Our goal was to test the hypothesis that -/low TS expression is associated with better objective response rate. Among the included studies, four of them reported a higher objective response in the TS -/low expression arm compared with the TS +/high expression arm [6,17,18,31]. While the rest four studies reported no statistically significant relationship between TS expression and response to chemotherapy [19,21,22,30]. We found that the objective response rate of patients with -/low TS expression was significantly higher than that in patients with +/high TS expression. We also conducted a trend that TS -/low predicted better median survival time and progression free survival time but without significant difference in patients receiving pemetrexed-containing chemotherapy.

Previous in vitro studies have shown that TS expression correlated with objective response of NSCLC treated with pemetrexed-containg chenmotherapy Ozasa et al. [15] documented that the expression level of the TS gene was significantly correlated with the concentration of pemetrexed for 50% cell survival (IC50) in 11 non-small cell lung cancer cell lines, suggesting up-regulation of the expression of the TS gene may have an important role in the acquired resistance to pemetrexed. Wu et al. found downing stream of TS gene may serve as new biomarkers for predicting responsiveness to pemetrexed [13]. Similar results were also reported by Chiappori in small cell lung cancer cell line [34]. In our meta analysis, the available data indicated that the quality of response to pemetrexed-containing chemotherapy was significantly higher in patients with -/low TS expression than those with +/high TS expression (OR = 0.46, 95% CI 0.29-0.72, *p* =0.0007). Interestingly, subgroup analysis based on whether platinum was conducted in their performance demonstrated -/low TS expression was associated a good objective response rate in platinum-containing subgroup. The knowledge gained from this subgroup analysis implys that treatment with pemetrexed plus cisplatin or carboplatin chemotherapy regimen may be more beneficial for -/low TS expression patient with NSCLC, compared with pemetrexed monotherapy regimen or pemetrexed plus gemcitabine.

The reason is thought to be that platinum may play an important role in the association of TS expression and overall objective response. Published study has shown that cisplatin binds to DNA and induces DNA cross-linking, which leads to DNA double- and single-strand breaks and causes cell death [35]. Pemetrexed inhibits DNA and RNA synthesis by impairing the activity of at least 3 enzymes, TS, GARFT and DHFR, in the folate metabolic pathway, a critical pathway for purine and pyrimidine synthesis [36]. In vitro sensitivity to platinum-derived drugs, cisplatin or carboplatin have been reported to be associated with expression of TS in human lung cancer [37]. Recent studies also demonstrated that cisplatin and pemetrexed may have a synergistic effect and low TS expression does not necessarily correlate with pemetrexed sensitivity [38]. Multiple mechanisms may underlie natural and acquired resistance to pemetrexed [37]. However, evaluation of a larger data set containing more prospective studies would provide greater confidence in efficacy between the two subgroups.

Different bio-methods were analyzed for their abilities to predict the treatment outcome. IHC detects TS expression at protein level, while RT-PCR assays at mRNA level. Several studies have reported some discrepancy between mRNA expression and protein expression for TS [24,39,40]. A recent research by Hou.et.al found that very high protein expression of TS determined by IHC correlates well with TS mRNA expression, while low protein expression of TS correlates poorly with TS mRNA expression [41]. In our meta-analysis, IHC seemed to be better than RT-PCR to predict objective responsive rate for lung cancer patients receiving pemetrexed-containing treatments. TS at the protein level help DNA synthesis and repair DNA double-strand breaks [42]. We think that’s the possible reason why IHC is better to predict the response rate than RT-PCR . Transcription, posttranscriptional regulation, translation, post-translation processing of the protein may also contribute to the difference. More prospective studies with large sample size are needed to further evaluate RT-PCR assay for TS detection and its power to predict chemotherapy sensitivity.

Intriguingly, although there was a trend that TS -/low predicted better median overall survival time and progression free survival time, the difference did not reached statistical significance [25]. Recent studies have reported that intratumoral TS expression was significantly related to the prognosis in patients with mesothelioma [43], gastric cancer [44] and colorectal cancer [45-47]. For NSCLC, low TS mRNA level was associated with a better PFS in stage I and II patients [48]. In our analysis, most patients available for evaluating median OS and PFS suffered from advanced or recurrent lung cancer and that may be the possible reason why no significant difference was found between patients with -/low an +/high expression.

The study has many limitations. Only eight studies are eligible for our meta-analysis and the sample size analyzed in each group was relatively small. However, this is the first and initial meta-analysis of assessment whether TS expression is associated with objective response in patients with NSCLC treated with pemetrexed-containing chemotherapy. More published studies will be helpful in clarifying whether this is a true association. There was no observed significant heterogeneity among the included studies (*I*^2^ = 25%), we further explored heterogeneity by conducting subgroup analyses. Although significant heterogeneity was not found either, heterogeneity in IHC subgroup was moderately high at 45%, which was mainly due to the diversity of regimen combinations and different populations. Publication bias is also a possible limitation because studies that report negative results are published less frequently than those reporting positive results or those consistent with prevailing theories [49]. However, we did not find that publication bias significantly influences our result of the meta-analysis.

## Conclusion

In conclusion, this meta-analysis provided us with evidence that -/low TS expression is associated with a higher objective response rate for NSCLC patients treated with pemetrexed-containg chemotherapy. Our results may be useful in matching NSCLC patients with suitable drugs and predict response rate to pemetrexed-containg chemotherapy as well as for the further investigation of random clinical trial on patients receiving platinum-based or pemetrexed-based chemotherapy.

## Competing interests

The authors have declared that no competing interests exist.

## Authors’ contributions

Conception and design: LW, RW and HC; Acquisition of data: all authors; Analysis and interpretation of data: all authors; Manuscript drafting: LW, JZ and HC; Manuscript revising: all authors; final approval of this version: all authors. All Authors read and approved the final manuscript.

## Pre-publication history

The pre-publication history for this paper can be accessed here:

http://www.biomedcentral.com/1471-2407/14/205/prepub
